# Thin and Dense Solid-solid Heterojunction Formation Promoted by Crystal Growth in Flux on a Substrate

**DOI:** 10.1038/s41598-017-18250-9

**Published:** 2018-01-08

**Authors:** Nobuyuki Zettsu, Hiromasa Shiiba, Hitoshi Onodera, Kazune Nemoto, Takeshi Kimijima, Kunio Yubuta, Masanobu Nakayama, Katsuya Teshima

**Affiliations:** 1 Center for Energy and Environmental Science, Shinshu University, 4-17-1 Wakasato, Nagano, 380-8553 Japan; 2 Department of Materials Chemistry, Shinshu University, 4-17-1 Wakasato, Nagano, 380-8553 Japan; 3 Institute for Materials Research, Tohoku University, 2-1-1 Aoba-ku, Katahira, Sendai, 980-8577 Japan; 4 Frontier Research Institute for Materials Science (FRIMS), Nagoya Institute of Technology, Gokiso, Showa, Nagoya, Aichi 466-8555 Japan; 5 Materials research by Information Integration Initiative (Mi2i), National Institute for Materials Science, Tsukuba, Japan

## Abstract

In this work, we demonstrate the direct growth of cubic Li_5_La_3_Nb_2_O_12_ crystal layer on the LiCoO_2_ substrate through the conversion of ultra-thin Nb substrate in molten LiOH flux. The initial thickness of the Nb layer determines that of the crystal layer. SEM and TEM observations reveal that the surface is densely covered with well-defined polyhedral crystals. Each crystal is connected to neighboring ones through the formation of tilted grain boundaries with Σ3 (2–1–1) = (1–21) symmetry which show small degradation in lithium ion conductivity comparing to that of bulk. Furthermore, the sub-phase formation at the interface is naturally mitigated during the growth since the formation of Nb_2_O_5_ thin film limits the whole reaction kinetics. Using the newly developed stacking approach for stacking solid electrolyte layer on the electrode layer, the grown crystal layer could be an ideal ceramic separator with a dense thin-interface for all-solid-state batteries.

## Introduction

All-solid-state rechargeable lithium ion batteries using oxide-based solid electrolytes have the potential to drastically improve the high energy, high power and safety of current lithium-ion batteries^1,2^. Thin-film type solid-state batteries have been prepared using thin film electrodes (micron- or submicron-order) prepared by physical vapor deposition approaches, including sputtering and pulsed laser deposition^3,4^. They have been commercialized and shown to be capable of >10,000 charge–discharge cycles^5^. To further increase the energy density of batteries, many researchers are currently focusing on bulk-type solid-state batteries. However, these batteries are known to have key performance problems. In particular, two issues are directly connected with the power density of batteries: the bonding of solid-solid hetero-interface between electrode and solid electrolyte, and thinning of the solid electrolyte separator^5–8^.

Various oxide electrolytes, such as perovskite-type Li_3x_La_2/3−x□1/3−2x_TiO_3_ (LLTO), LISICON, and NASICON have been widely investigated. In particular, garnet-type Li_7−*x*_La_3_A_2−*x*_^4+^B_*x*_^5+^O_12_ (A = Zr, B = Nb, Ta) derivatives are considered promising candidate materials due to their high Li^+^ conductivity (around 10^−4^ S cm^−1^ at room temperature) and wide electrochemical window. They are neither oxidized nor reduced in a wide voltage range^9–13^. The control of balance in the Li^+^-occupying sites of tetragonal-24d/octahedral-96h is found to be primarily responsible for both the Li^+^ conductivity and stability under air moisture. Furthermore, Narayanan *et al*. demonstrated that incorporating external defects, such as replacing Y with Nb (Li_5+2x_La_3_Nb_2−x_Y_x_O_12_), enhanced the Li^+^ conductivity by two orders of magnitude in Li_5_La_3_Nb_2_O_12_^14^.

It is generally believed that practical all-solid-state lithium ion batteries should ideally be fabricated by a solid-phase process for achieving dense solid interfaces. Since the architectures at interface, including both material density and purity, strongly affect the electrochemical performance and interfacial resistance, we need to consider how to bond interfaces of polycrystalline ceramic powders while suppressing the side reactions that increase the interfacial resistance^15,16^. Existing literatures on solid-solid homo- and heterojunction formation suggest that lowering the reaction temperature is effective for suppressing the sub-phase formation, promoted by the introduction of a buffer layer into the interface^8^ or using new discontinuous heating processes^5^. However both these conventional and new heating process under historical powder-based process often have technical limitations on the thinning of dense sintering bodies, as comparing to that of vacuum phase processing. Since the densification reaction is caused by diffusion of chemical species in solids based on the chemical potential gradient formed at the boundary of the contact interface between individual particles, the filling of the raw material powder closely in a crucible at the preliminary forming stage before sintering effectively form a dense sintered body densely. Furthermore, the use of raw materials having a non-uniform shape also makes it difficult to effectively fill the voids.

Very recently, solid electrolyte thin films, fabrication by using pulsed laser deposition (PLD)^17,18^, radio-frequency magnetron sputtering^19^, sol-gel process^20^, and aerosol deposition^21^ have been reported. Even though it was expected that such solid electrolyte thin film provides low electrical resistance and increasing volumetric energy density for all-solid-state battery, their lithium ion conductivities at room temperature were in the range of 10^−7^–10^−5^ S·cm^−1^, which is nearly two digits lower than sintered pellets samples. These film fabrication methods are efficient for fabricating uniform film and controlling the film thickness, however in some cases, it is difficult to control elementary composition and microstructures. Especially, except for epitaxial growth on a single crystal substrate^18^, there is no effective way for controlling the crystal growth orientation and grain boundary structures. Moreover, increasing substrate temperature during deposition and/or post-annealing should be needed to obtain well-crystallized films. Since high temperature treatments may lead to undesired reactions at the interface or uncontrolled diffusion between electrode and solid electrolyte, these methods are not recommended in some cases for fabricating all-solid-state batteries.

Based on the above considerations, here we demonstrate the formation of cubic phase stoichiometric Li_5_La_3_Nb_2_O_12_ crystal layers that are less than 30-μm thick, via the conversion of ultra-thin Nb substrate in a LiOH flux. We further discuss the reaction mechanism and crystallographic characteristics related to the crystal layer formation based on cross-sectional electron microscopic analysis. In addition, heterojunction formation between Li_5_La_3_Nb_2_O_12_ and LiCoO_2_ ceramics (which is the most popular cathode active material) was performed through a similar approach.

## Results

### Growth of Li_5_La_3_Nb_2_O_12_ crystal layer

**Figure 1:**
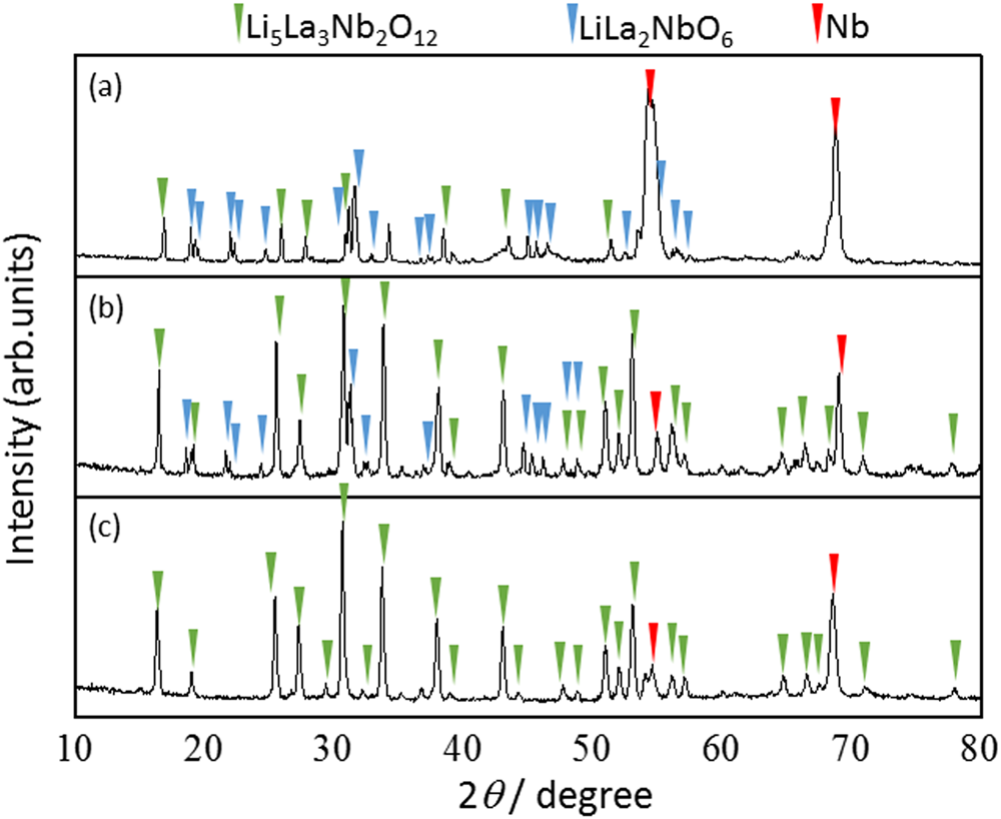
Time-dependent XRD patterns of the Nb substrate during reaction in a molten LiOH flux with La_2_O_3_ at 500 °C in air: (**a**) 10 min, (**b**) 60 min, and (**c**) 600 min.

**Figure 2:**
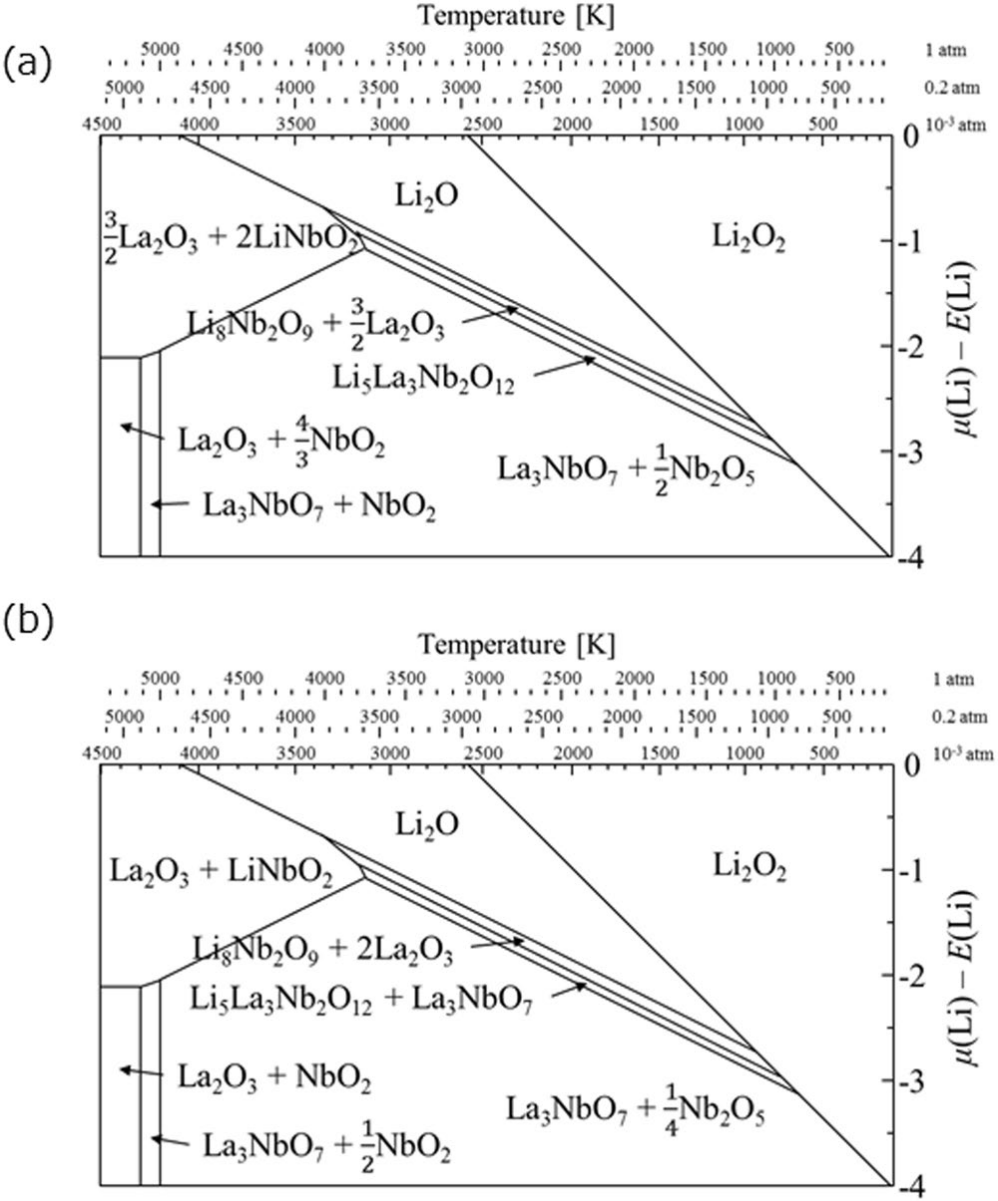
Phase diagram of Li-La-Nb-O system with respect to the chemical potential of Li and temperature with different molar ratios of La/Nb: (**a**) La/Nb = 1.5 and (**b**) La/Nb = 2.

The Li_5_La_3_Nb_2_O_12_ crystal layers were prepared on a Nb substrate by the flux growth method using a LiOH flux, in which LiOH·H_2_O and La_2_O_3_ powders reacted with the Nb substrate. Time-dependent XRD measurements were performed. As shown in Fig. 1, the formation of Li_5_La_3_Nb_2_O_12_ was initiated at 500 °C for 1 min. The diffraction lines were assigned to cubic phase Li_5_La_3_Nb_2_O_12_, perovskite LiLa_2_NbO_6_, and Nb, respectively. Perovskite LiLa_2_NbO_6_ is considered an intermediate phase which lacks Li and La compared to the stoichiometric composition of Li_5_La_3_Nb_2_O_12_. In fact, there are no reports on the formation of stable LiLa_2_NbO_6_ phase during the synthesis of Li_5_La_3_Nb_2_O_12_. Neither is the LiLa_2_NbO_6_ phase found in the ternary phase diagram of Li_2_O-La_2_O_3_-Nb_2_O_5_ system^22^. We further carried out computational studies to predict the LiLa_2_NbO_6_ phase formation in Li_2_O-La_2_O_3_-Nb_2_O_5_ system under various temperatures and pressures. As shown in Fig. 2 (a),(b), the LiLa_2_NbO_6_ phase was not formed when the starting materials were mixed in either stoichiometric or non-stoichiometric compositions. All these results indicate that the LiLa_2_NbO_6_ phase is not thermodynamically stable.

The formation of Li_5_La_3_Nb_2_O_12_ progressed with increasing holding time. The cubic phase Li_5_La_3_Nb_2_O_12_ was obtained as a single phase after 10 h. It is interesting to note that the Li_5_La_3_Nb_2_O_12_ phase was the major product during the early stages of the reaction, despite the unreacted La_2_O_3_ and LiOH remaining in the flux. Furthermore, no diffraction pattern assignable to niobium oxides such as Nb_2_O_5_ was detected during the growth process. In contrast, *in-situ* XRD measurement in atmosphere without using the LiOH flux revealed that the metal Nb substrate surface was oxidized to Nb_2_O_5_ at 500 °C (Fig. S1). Very similar results were observed in our previous reports that describe the formation of hollow-structured LiCoO_2_, LiMn_2_O_4_, and LiNi_0.5_Co_0.2_Mn_0.3_O_2_ from the conversion of electrodeposited Co, Mn, and stacked Ni/Co/Mn dots in molten salts at high temperatures, respectively^23,24^. Based on these results, the oxide formation on the Nb substrate could limit the reaction kinetics, since the amount of dissolved oxygen is normally small in the molten salts.

**Figure 3:**
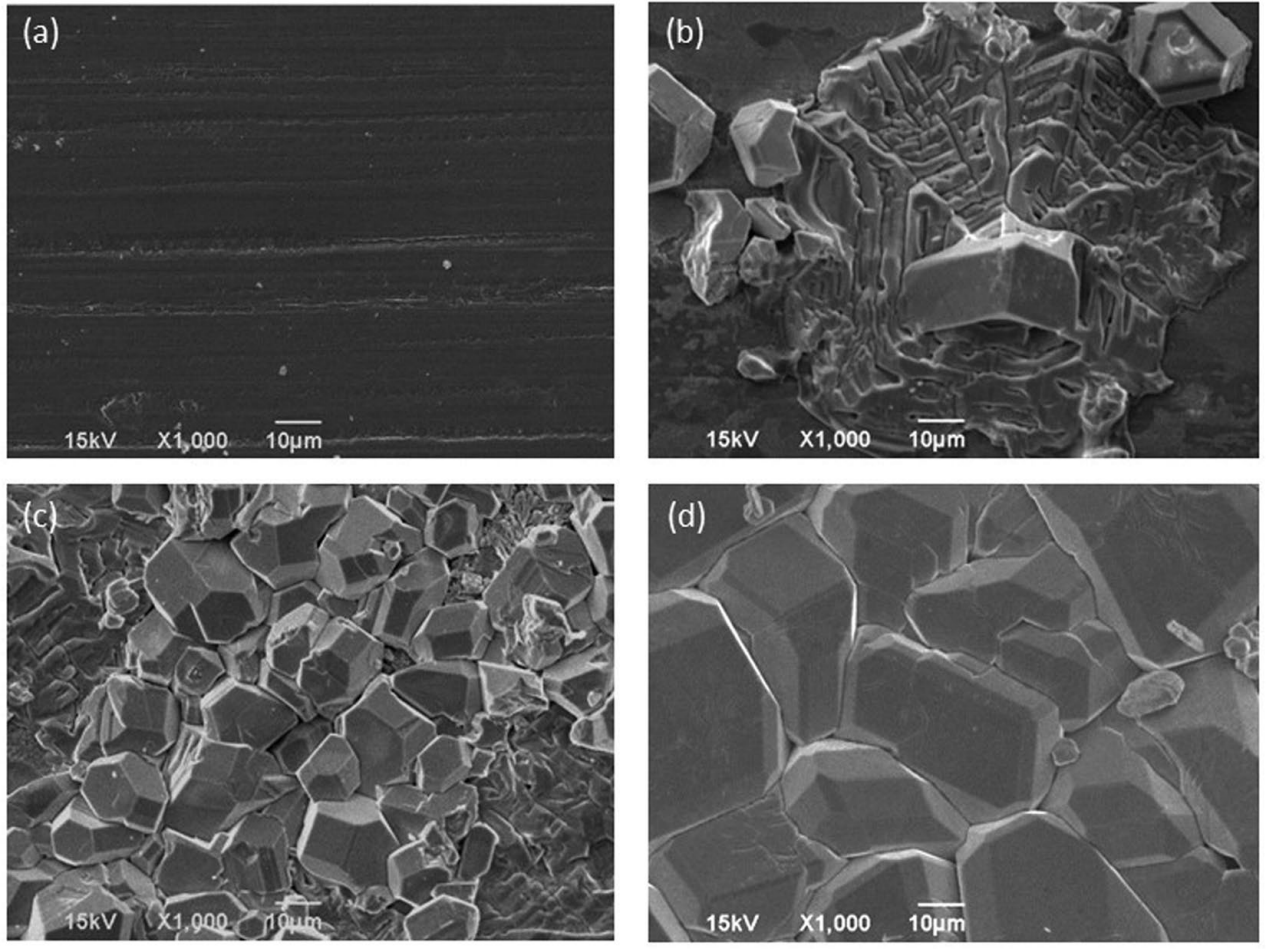
Time-dependent SEM images of the Nb substrate during reaction in a molten LiOH flux with La_2_O_3_ at 500 °C in air: (**a**) as-purchased Nb substrate, after (**b**) 10 min, (**c**) 60 min, and (**d**) 600 min.

Next, we carried out time-dependent SEM observations coupled with XRD measurements to further understand the reaction and growth mechanisms (Fig. 3). Two different kinds of morphologies, the spirally-grown flat shape and the 3D polyhedral shape, were formed on the Nb substrate after heating at 500 °C for 1 min in molten LiOH. The flat-shaped crystal layer developed laterally as the reaction continued, and the polyhedral crystals multiplied simultaneously. Finally, after 10 h, the surface of Nb substrate was completely covered with polyhedral crystals with well-defined faces. The individual polyhedral crystals have a wide range of diameters, with the mean diameter ~30 μm. The face angle analysis suggests that the crystal surface was dominantly surrounded by larger {211} and smaller {110} faces. Such crystallographic features dovetail with those of Li_5_La_3_Nb_2_O_12_ single crystals that were homogeneously grown from LiOH flux (with LiOH·H_2_O, La_2_O_3_, and Nb_2_O_5_ powders reacting in a crucible)^25^.

**Figure 4:**
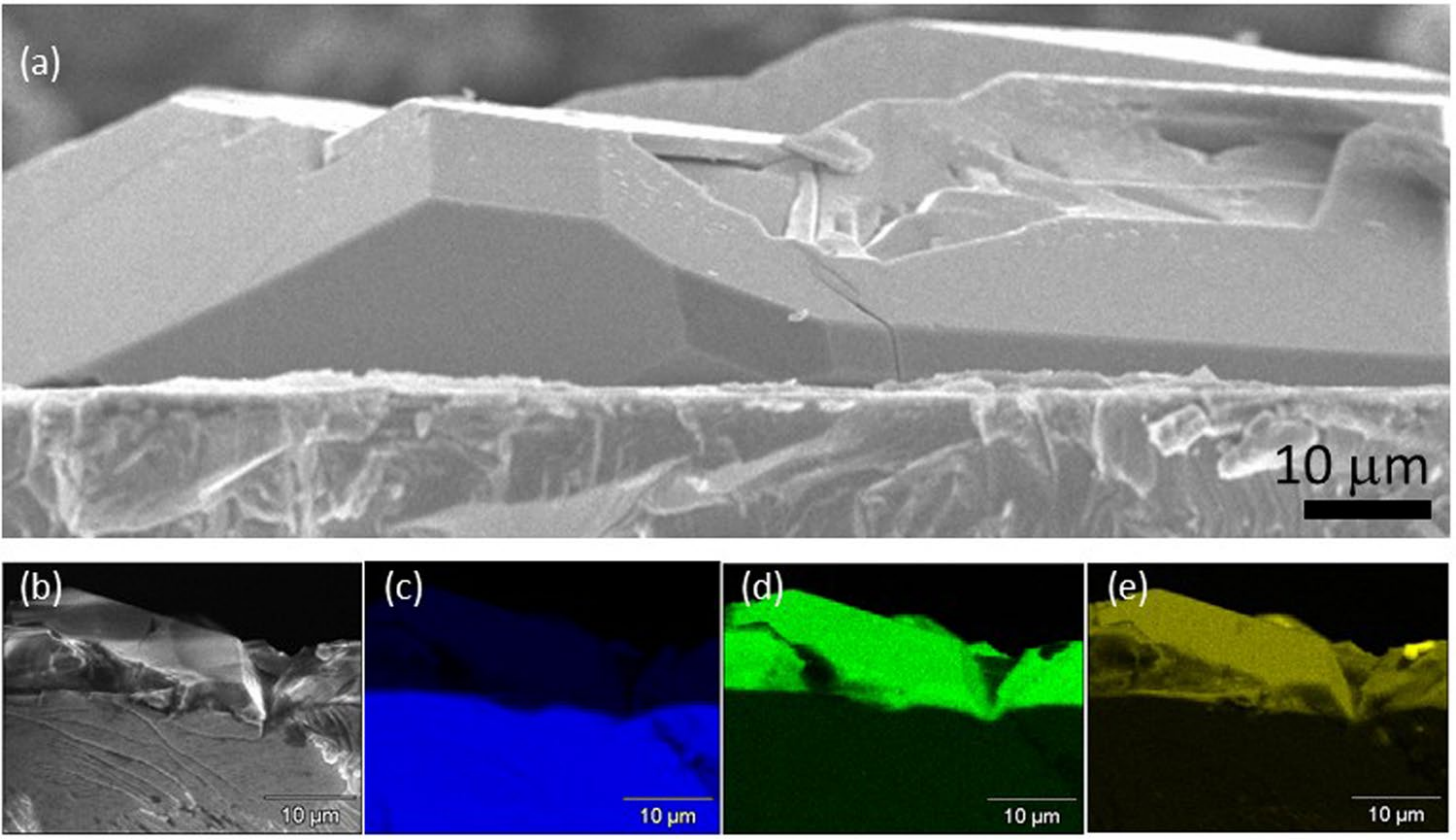
(**a**) Cross-sectional SEM image and (b-e) SEM-EDS elemental mapping of the Li_5_La_3_Nb_2_O_12_ crystal layer on Nb substrate: (**b**) SEM-image, (**c**) Nb, (**d**) La, and (**e**) O.

Cross-sectional SEM observation revealed that the polyhedral crystals grew directly from the Nb surface (Fig. 4). There were no detectable defects such as voids, spaces, and twins at the interface. The spirally grown flat crystalline layer was hardly observed at the interface. This result strongly suggests the possible limiting reaction suggested above. If the formation of LiLa_2_NbO_6_ through the reaction of LiOH·H_2_O and La_2_O_3_ powders with Nb_2_O_5_ layer limits the whole reaction, LiLa_2_NbO_6_ and/or Nb_2_O_5_ layer should remain at the interface. Thus, the thickness of Li_5_La_3_Nb_2_O_12_ crystal layer will be determined by the initial thickness of the oxidized Nb layer prior to the reaction. Indicating that the self-diffusion rate of Nb is not much faster than the diffusion of O_2_ in the molten LiOH flux. If the self-diffusion rate of Nb is much faster than the diffusion of O_2_ in the molten LiOH flux, the anisotropic atomic motion at the boundary layer, which is a consequence of the different diffusion rates, will prompt the formation of voids and spaces at the interface.

**Figure 5:**
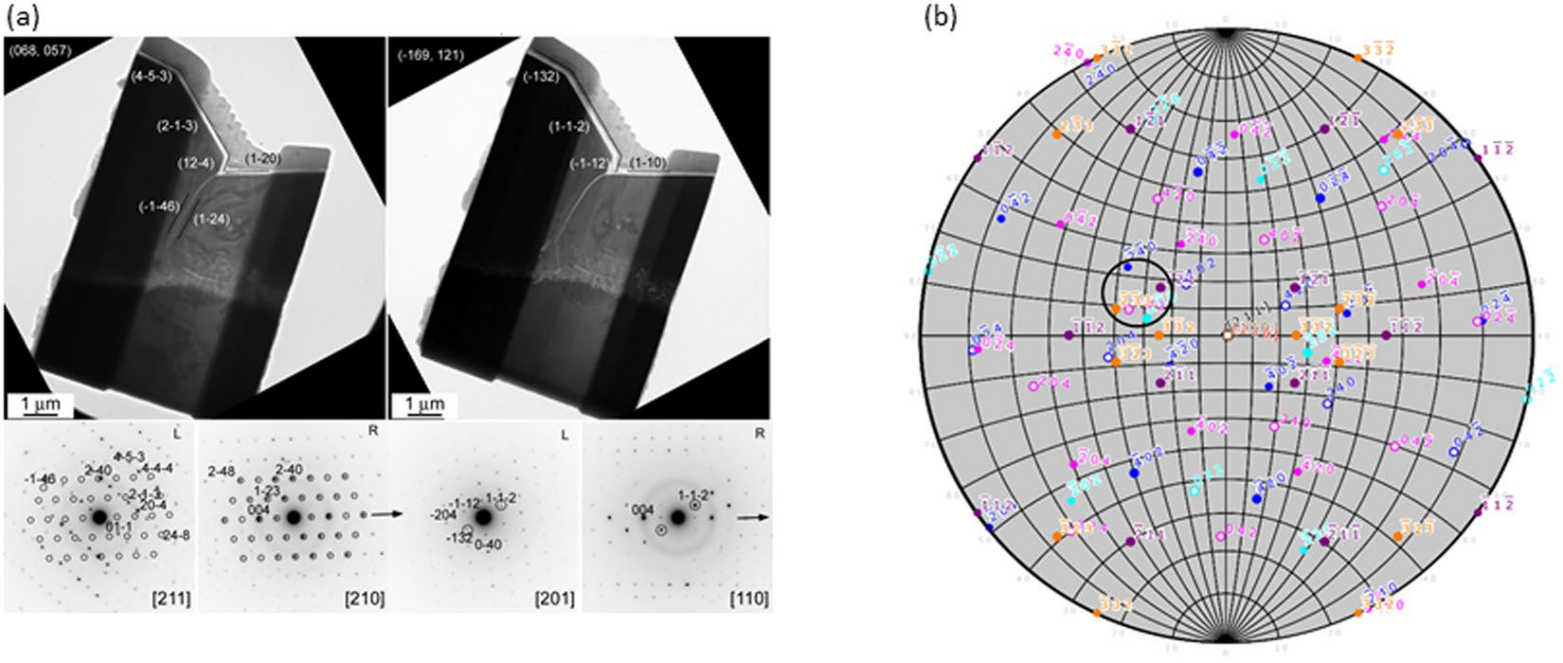
(**a**) Cross-sectional TEM images and SAED patterns of FIB-milled thin Li_5_La_3_Nb_2_O_12_ crystal layer. “L” and “R” in the diffraction pattern mean diffraction patterns observed from the left and right grains in the TEM image, respectively. (**b**) A stereo diagram based on cross-sectional TEM observations of the sample.

Cross-sectional SEM observation also indicates that each individual crystal seems to connect to neighboring crystals through the formation of tilted grain boundaries. In order to characterize their 3D crystallographic orientation in the selected area near the grain boundaries, we performed cross-sectional TEM observations of the sliced Li_5_La_3_Nb_2_O_12_ crystal layer which was created using FIB-milling. The cross-sectional TEM images and SAED patterns are shown in Fig. 5(a). “L” and “R” in the diffraction patterns mean patterns observed from the left and right grains in the TEM image, respectively. All the images and diffraction patterns were taken from the same sample. These are images tilted 24.3°, with the horizontal arrow direction (the [001]* axis of R grain) in the diffraction pattern of the R region as the tilt axis. Therefore, the angle between these two directions can be estimated as 24.1°. The image and diffraction pattern shown on the right side are taken from the same position with 24.3° tilting around the [00 l] axis of the R region. The analysis of these diffraction patterns revealed that the [201] direction (in L grain) and the [110] direction (R grain) are almost in agreement. From the above observations, it became clear that the crystal orientation is aligned three-dimensionally in this crystal region. Figure 5(b) shows a stereo diagram based on a number of cross-sectional TEM observations for the FIB-milled Li_5_La_3_Nb_2_O_12_ crystal layers. Superimposed on it are the stereo diagrams for the case where the incident [211], [110], and [210] incidence as the poles from cross-sectional TEM observation. It can be seen that the crystal planes {120} and {112}, which appeared on the surface at each incidence angle, are close to each other and enclosed by the circle in the figure. This result suggests that there may be a direct correlation with crystal orientation of crystal habit which appears in different grains.

We have reported the Li^+^ conductivity of the sinter bodies prepared from the Li_5_La_3_Nb_2_O_12_ crystals of which it was grown from LiOH flux under same recipe described in this paper^25^. The Li^+^ conductivity was of 5 × 10^−6^ S·cm^−1^ at room temperature, as evaluated by conventional electrochemical impedance spectroscopy. Unfortunately, reproducible data on the Li^+^ conductivity for the Li_5_La_3_Nb_2_O_12_ crystal layers have not yet to acquire because any symmetrical cells, Li| Li_5_La_3_Nb_2_O_12_ crystal layer|Li for non-blocking geometry and Au | Li_5_La_3_Nb_2_O_12_ crystal layer |Au for blocking geometry were not obtained at this time. It is difficult to make free-standing film required for making symmetrical cells due to the strong adhesion strength to the Nb substrate. We are now going to demonstrate AC impedance measurement of the Li_5_La_3_Nb_2_O_12_ crystal layers by using micro-probe technique^26^. We will report our new findings near feature in elsewhere soon.

### Molecular dynamics simulation to evaluate the lithium ion conductive characteristics at the tilted grain boundary of Σ3 (2–1–1) = (1–21)

**Figure 6:**
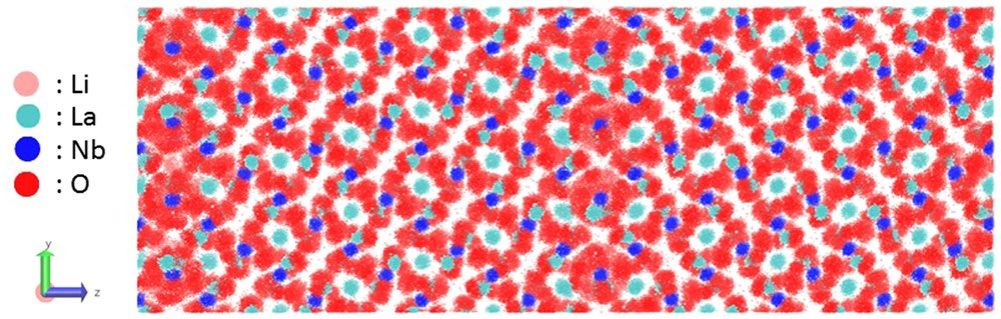
Trajectories of the Li, La, Nb, and O framework atoms obtained for Σ3 (2–1–1) = (1–21) at a temperature of 1300 K.

**Figure 7:**
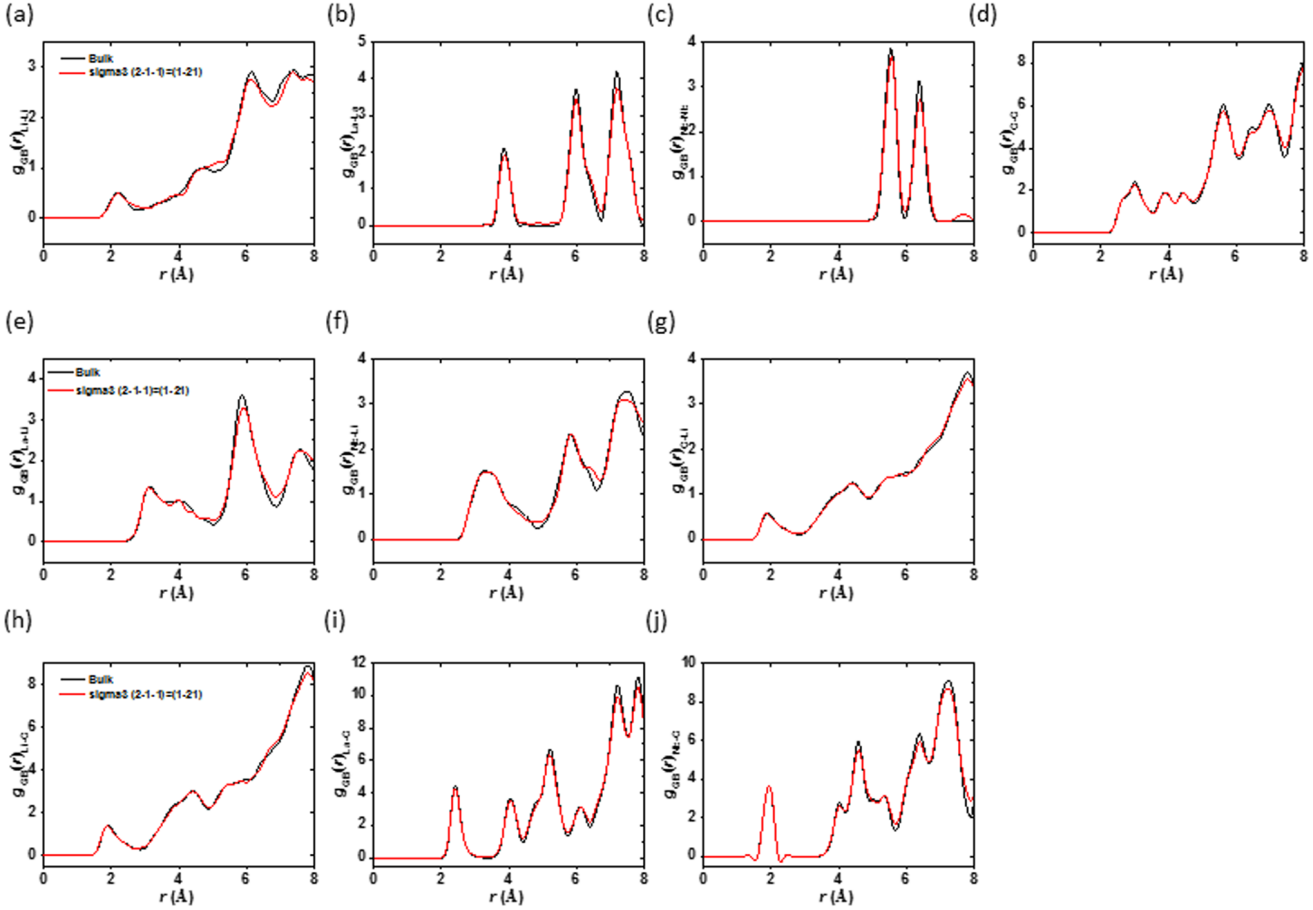
RDFs for the Li, La, Nb, and O interactions in the bulk (black) and at the tilted grain boundary Σ3 (2–1–1) = (1–21) model (red): (**a**) Li-Li, (**b**) La-La, (**c**) Nb-Nb, (**d**) O-O, (**e**) La-Li, (**f**) Nb-Li, (**g**) O-Li, (h) Li-O, (**i**) La-O, and (**j**) Nb-O.

**Figure 8:**
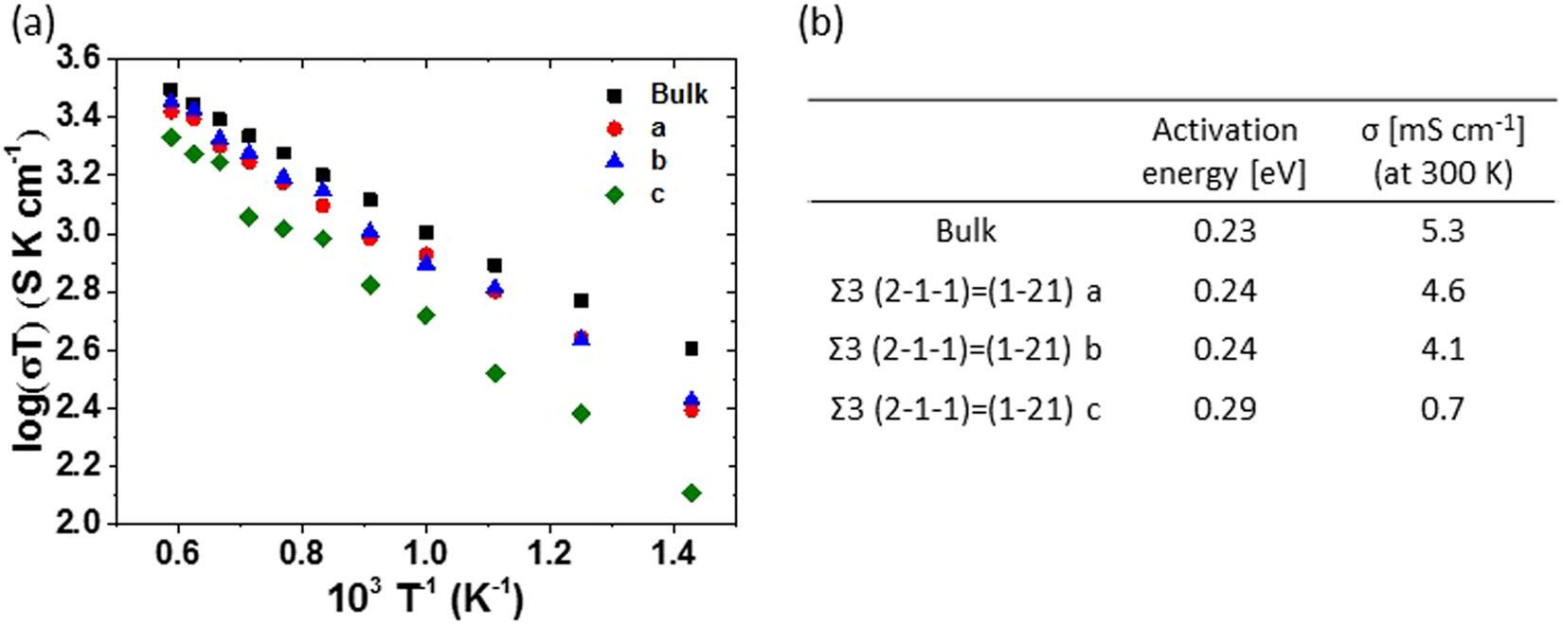
(**a**) Arrhenius plots of the Li^+ ^conductivity calculated for isotropic 3D Li diffusion path in the bulk garnet Li_5_La_3_Nb_2_O_12_ along the *a*, *b*, and *c* axes. (**b**) Fitted Li^+^ conductivity at 300 K derived from the MSD analysis.

A stoichiometric equilibrium atomic model was calculated by using MD simulation for understanding the atomic arrangement and determining the Li ion conductivity at the tilted grain boundary of Σ3 (2–1–1) = (1–21), as a one representative grain boundary model. Figure 6 displays the trajectories with respect to the available La (light brown), Nb (blue), O (red), and Li (yellow) crystallographic sites and calculated via MD simulations for a temperature of 1300 K and duration of 500 ps. Trajectories of the framework atoms revealed that there was hardly any migration of the La, Nb, and O atoms. In addition, Fig. 7 displays the radial distribution function (RDF) plots obtained for the Li-Li, La-La, Nb-Nb, and O-O interactions in the bulk and at the grain boundary, from the MD simulations conducted at 700 K and total duration of 500 ps. The results in Fig. 8 suggest no remarkable differences among the La, Nb, and O sites as compared to the Li atoms, indicating that only small atomic rearrangements were present at the crystallographic sites of the Σ3 (2–1–1) = (1–21) titled grain boundary model. Interestingly, the grain boundary formation energy was estimated as 0.23 J m^−2^, which is markedly smaller than that of cubic-Li_7_La_3_Zr_2_O_12_ (0.52 J m^−2^)^27^. It indicates that the tilted grain boundary represented by the Σ3 (2–1–1) = (1–21) models is one of the most stable grain boundaries in the Li_5_La_3_Nb_2_O_12_ system. This computationally predicted trend is consistent with the thermodynamically stable faces of the Li_5_La_3_Nb_2_O_12_ crystals grown from a molten LiOH flux^25^.

Figure 8(a) describes the computationally predicted apparent Li^+^ conductivities of the bulk and the Σ3 (2–1–1) = (1–21) models, including the average and separate conductivities along the a, b, and c axes. It should be noted that the Li^+^ conductivities of the utilized grain boundary model included bulk conductivities, because of the presence of bulk regions in the model. The corresponding Li^+^ diffusion coefficients and conductivities at a specified temperature can be evaluated from the slopes of MSD plotted against time and the Nernst-Einstein equation, respectively. The values of the fitted Li ionic conductivity at 300 K, which were obtained from the Arrhenius plots for the models, are summarized in Fig. 8(b). The computationally predicted bulk Li^+^ conductivity is in good agreement with data in previous reports^28^. However, it was almost two orders of magnitude higher than the experimental value evaluated by electrochemical impedance spectroscopy for the pelletized samples.

The grain boundary conductivity in the cubic Li_5_La_3_Nb_2_O_12_ is lower than the bulk one, regardless of the orientations. In particular, the conductivities along the *c*-axis (perpendicular to the grain boundary) are the lowest, being one order of magnitude lower than the bulk value. To our best knowledge, this study reports for the first time that the conductivity along the direction perpendicular to the grain boundaries primarily contributes to the reduction in the total ion conductivity. Most previous electrochemical impedance spectroscopic studies of garnet-based solid electrolytes could not distinguish the tilted grain boundary contribution driven by atomic arrangements within the overall material resistance, thus underestimating the experimental bulk conductivity. Furthermore, the elemental substation of Nb with Zr, leading to form Li_7_La_3_Zr_2_O_12_ was known to enhance bulk Li^+^ conductivity through changing the Li^+^ occupancy at 24d/96 h sites^12^, we newly discovered that the substitution made significant degradation of the grain boundary conductivity at the Σ3 (2–1–1) = (1–21). Our molecular dynamics simulation to the Li^+^ conductivity in Li_7_La_3_Zr_2_O_12_ revealed that the fitted Li ionic conductivity at 300 K were ca. 38.1 mS·cm^−1^ in bulk and 0.7 mS·cm^−1^ in the grain boundary. The grain boundary conductivity decreased nearly three orders than that of bulk. It was quite different from the Li_5_La_3_Nb_2_O_12_ system. We believe our results here strongly suggest that the observed discrepancy between the experimental and computational Li^+^ conductivities may be attributed to the effect of the grain boundary resistance on the atomic arrangement.

### Growth of Li_5_La_3_Nb_2_O_12_ crystal layer on LiCoO_2_ ceramics

**Figure 9:**
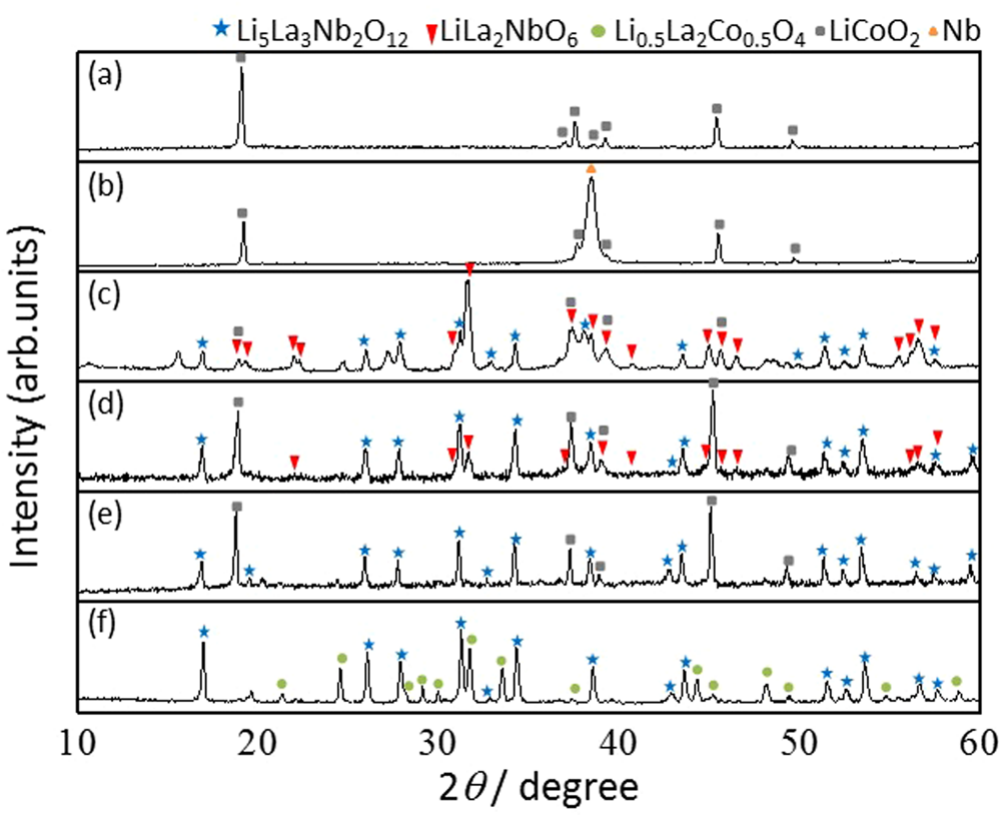
Time-dependent XRD patterns of LiCoO_2_ ceramic substrates: (a) as-purchased, (**b**) after Nb deposition, (**c**) held at 500 °C for 1 h, (**d**) 5 h, (**e**) 10 h, and (**f**) 20 h.

**Figure 10:**
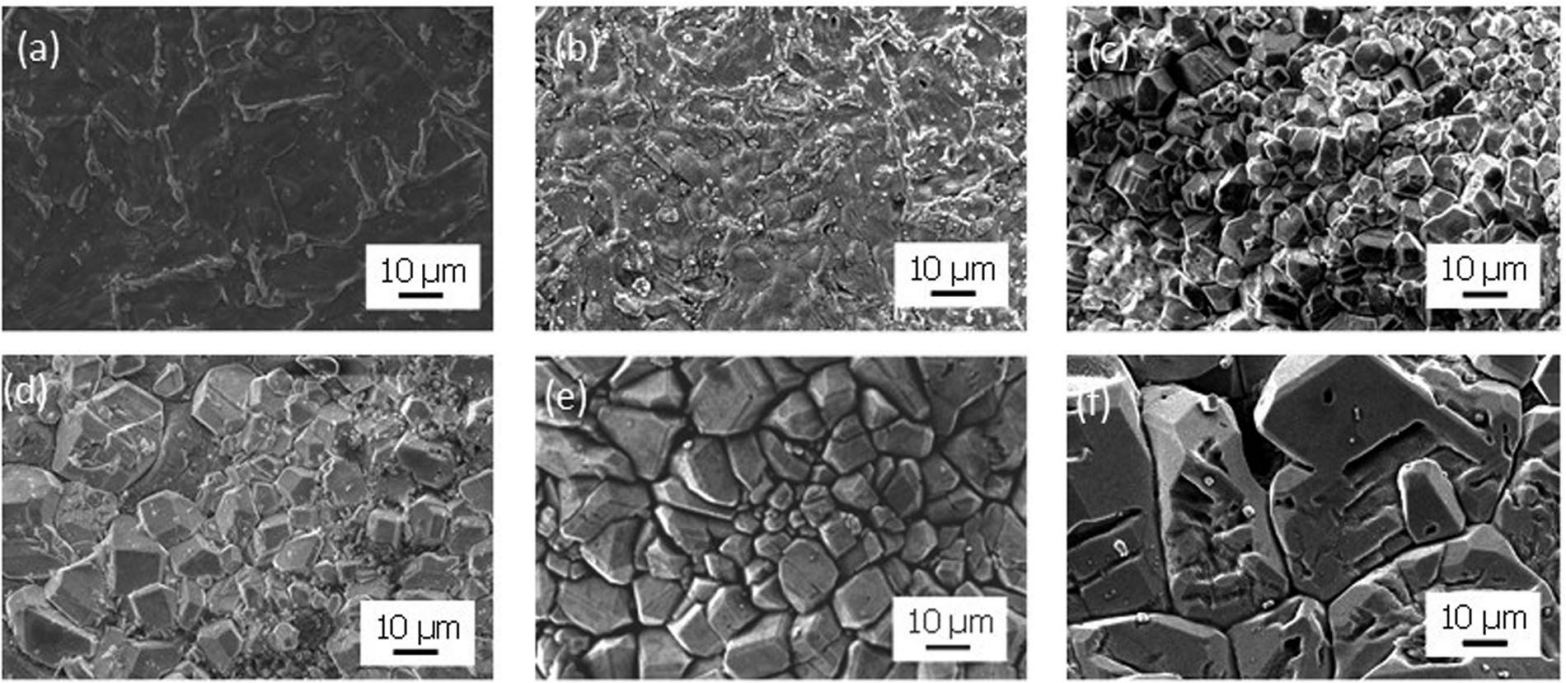
Time-dependent SEM images of LiCoO_2_ ceramic substrates: (**a**) as-purchased, (**b**) after Nb deposition, (**c**) held at 500 °C for 1 h, (**d**) 5 h, (**e**) 10 h, and (**f**) 20 h.

We applied the same methodology to stack the Li_5_La_3_Nb_2_O_12_ crystal layer on LiCoO_2_ ceramics through the direct growth from the LiCoO_2_ surface. Sputtered thin Nb film was deposited prior to the growth, and was used as the Nb source instead of a metallic Nb substrate. The LiCoO_2_ ceramic surface turned to a brilliant color after the deposition. Time-dependent XRD holding at 500 °C reveals that the perovskite LiLa_2_NbO_6_ phase was dominantly formed during the initial stage of reaction (Fig. 9). This phase was sequentially transformed into cubic Li_5_La_3_Nb_2_O_12_ as the reaction time increased. The cubic Li_5_La_3_Nb_2_O_12_ phase with garnet framework was eventually obtained as a single phase after 10 h, without any sub-phase formation. Note that an excessively long reaction time was found to promote the formation of byproduct phases, such as Li_0.5_La_2_Co_0.5_O_4_, at the heterophase interface. This is thought to be due to the mutual diffusion of Co in LiCoO_2_ and La in Li_5_La_3_Nb_2_O_12_ at the interface. Time-dependent SEM observation was used to characterize the growth and development of Li_5_La_3_Nb_2_O_12_ crystal layer on the LiCoO_2_ surface. As shown in Fig. 10(a),(b), the initial surface morphology was drastically changed with increasing reaction time. Numerous polyhedral crystals having well-defined faces gradually covered the surface. Prolonged reaction time promoted the growth of the individual crystals, and their size distribution became simultaneously broader. These time-dependent crystal growth characteristics in molten LiOH flux strongly support that the crystal growth is driven by Ostwald’s ripening. The whole surface was covered by the Li_5_La_3_Nb_2_O_12_ crystal layer after 10 h (Figs 9(e) and 10(e)). However, further reaction caused voids to form on the crystal surface due to the Li_7_La_3_Nb_2_O_12_ phase formation. In addition, many large pin-holes were formed at the grain boundaries, driven by over growth of the Li_5_La_3_Nb_2_O_12_ crystal (Fig. 10(f)). Interestingly, the formation of pin-holes and the Li_0.5_La_2_Co_0.5_O_4_ phase occurred simultaneously.

Cross-sectional SEM combined with EDS mapping clearly showed that the elemental distribution in Co is very different from those of Nb and Zr (Fig. S2), meaning that the ultra-thin Nb film, sequentially formed Nb_2_O_5_ layer, and LiLa_2_NbO_6_ and Li_5_La_3_Nb_2_O_12_ crystal layers mitigated the sub-phase (Li_0.5_La_2_Co_0.5_O_4_) formation during the growth. M. Bitzer *et al*. reported that the formation of unwanted Li_0.5_La_2_Co_0.5_O_4_ phase at the LiCoO_2_/Li_7_Li_3_Zr_2_O_12_ interface of which it appears that La diffused from the Li_7_Li_3_Zr_2_O_12_ electrolyte into the LiCoO_2_ cathode during calcination. They further claimed that the Li_0.5_La_2_Co_0.5_O_4_ phase formation at the electrode/electrolyte interface resulted in significant Li^+^ conductivity fading^29^. We believe that the fabrication route which achieve the suppression of sub-phase formation at the hetero-interface will be an ideal avenue to produce thin and dense ceramic separators. In fact, no electrical short-cut was observed in the stacking structures of Li_5_La_3_Nb_2_O_12_ crystal layer and LiCoO_2_ ceramics after the deposition of In metal as a counter electrode (an anode).

## Discussion

We have studied the direct growth of cubic Li_5_La_3_Nb_2_O_12_ and related compounds on solid substrates. The conversion of Nb substrate in molten LiOH flux formed crystal layers with well-defined faces, and each individual crystal is connected to neighboring ones. Cross-sectional TEM observation coupled with SAED pattern analysis implied the formation of tilted grain boundaries that are composed of [211], [110], and [210] faces. We further found that the ultra-thin Nb film naturally mitigates sub-phase formation at the interface during the growth process, since the formation of niobium oxides on the substrate limits the reaction kinetics. We believe that our approach having robustness against side reactions at the interface could possibly lead to the production of ideal ceramic separators with a thin and dense interface. The LiCoO_2_ ceramics used in this study (1 mm in thickness) was too thick for solid batteries and could not perform as such at this stage. However, as long as the electrode layer can be made as thin as 100 μm, the stacking layer will operate as a solid battery.

## Materials and Methods

### Materials

Metal Nb substrate (Nilaco Corporation) La_2_O_3_, and LiOH·H_2_O (Wako Pure Chemical Industries) were used for the preparation of the Li_5_La_3_Nb_2_O_12_ crystal layer. The Nb substrate was cut into small pieces with dimension of 10 mm × 15 mm prior to use. LiCoO_2_ ceramics (*φ* = 13 mm × 1 mm thick) were purchased from KCM Corporation. All commercial materials were used without further purification.

### Characterization

The morphological and elemental mapping analyses of the grown crystal layers were carried out using field-emission scanning electron microscopy (FE-SEM) coupled with an analyzer for energy dispersive X-ray spectroscopy (EDX) (JEOL, JSM-7600F). An acceleration voltage of 15 kV was used. Pass energy was controlled to be The phases and structures of the crystals were identified using X-ray diffraction (XRD) analysis with Cu Kα radiation. The X-ray diffractometer (RIGAKU, Miniflex II) was operated at 30 kV and 20 mA, with 2*θ* = 10–80°. For the cross-sectional TEM observation and crystallographic orientation analysis of the Li_5_La_3_Nb_2_O_12_ crystal layer on Nb substrate, thin specimens were prepared by a focused ion beam apparatus with Ga gun (FIB, JIB-4000, JEOL). Selected area electron diffraction (SAED) patterns and bright-field TEM images were obtained using a 200kV electron microscope (TOPCON EM-002B).

### Growth of Li_5_La_3_Nb_2_O_12_ crystal layer through conversion of metallic Nb substrate in molten LiOH flux

We applied similar recipe of the flux growth of polyhedral shaped Li_5_La_3_Nb_2_O_12_ single crystals the crystal layer formation^25^. La_2_O_3_ (0.305 g) and LiOH·H_2_O (0.295 g) were mixed in an alumina crucible 30 mL in volume for 30 min. To adjust the solute concentration to 5 mol%, 2.431 g of LiOH·H_2_O powder was further added as a flux. A Nb substrate was gently placed a the mixture and then heated to 500 °C at a rate of 500 °C·h^−1^ in an electric furnace. The substrate was cooled to 300 °C at a rate of 100 °C·h^−1^, and then naturally cooled to room temperature in the furnace. The substrate was separated from the mixture by washing with warm water for three times to remove the remaining flux, completely. Finally, the substrate was dried at 60 °C in air for 10 h.

### Molecular dynamics simulation of the phase diagram of Li-La-Nb-O system

The Vienna *ab initio* simulation package^30,31^ was used with the generalized gradient approximation (GGA-PBE sol) + *U* ^32^ and projector-augmented wave methods^33^. For the GGA + *U* calculations, the *U* value for the *d*-orbitals of Nb was set to 1.5 eV, based on previous reports^34^. An energy cutoff of 500 eV and a *k*-point mesh were chosen, so that the product of the number of *k*-points and the number of atoms in the unit cell was greater than 1000. To construct the phase diagram of the Li-La-Nb-O system, we obtained the possible compositions from Inorganic Crystal Structure Database (ICSD) and optimized the structures of related oxides (in this case they are Li_2_O, Li_2_O_2_, La_2_O_3_, NbO_2_, Nb_2_O_5_, LiNbO_2_, LiNbO_3_, LiNb_3_O_8_, Li_3_NbO_4_, Li_8_Nb_2_O_9_, LaNbO_4_, LaNbO_7_, LiLaNb_2_O_7_, LiLa_2_NbO_6_, and Li_5_La_3_Nb_2_O_12_). The total energy, *E*_*total*_ (X), obtained by DFT calculations was assumed as the sum of chemical potentials *μ*(Y): *E*_*total*_ (Li_5_La_3_Nb_2_O_12_) = 5(Li^+^) + 3(La) + 2(Nb) + 12(O). The chemical potential of oxygen molecule was set as zero in the horizontal axis, and that of lithium metal was set as zero in the vertical axis. The energy correction for O_2_ molecules was used for all calculations as reported by Wang *et al*.^35^. At the boundaries between two phases, the chemical potentials of each atom were assumed to be equal in the two phases. The boundaries between two phases were calculated by simultaneous equations. For example, the boundary between Li_5_La_3_Nb_2_O_12_ and (La_3_NbO_7_ + Nb_2_O_5_) phases was calculated by the following equations:

1 $$\begin{array}{c}{E}_{total}({{\rm{Li}}}_{{\rm{5}}}{{\rm{La}}}_{{\rm{3}}}{{\rm{Nb}}}_{{\rm{2}}}{{\rm{O}}}_{{\rm{12}}})=5\,\mu ({\rm{Li}})+3\,\mu ({\rm{La}})+2\,\mu ({\rm{Nb}})+12\,\mu ({\rm{O}})\\ {E}_{total}({{\rm{La}}}_{{\rm{3}}}{{\rm{NbO}}}_{{\rm{7}}})=3\,\mu ({\rm{La}})+\mu \,({\rm{Nb}})+7\,\mu ({\rm{O}})\\ {E}_{total}({{\rm{Nb}}}_{{\rm{2}}}{{\rm{O}}}_{{\rm{5}}})=2\,\mu ({\rm{Nb}})+5\,\mu ({\rm{O}})\end{array}$$

We obtained the phase diagram of Li-La-Nb-O system by calculating all the possible simultaneous equations similar to the ones given above. Note that the molar ratio of La/Nb is fixed as 2 and 1.5 in the phase diagrams. We convert the chemical potential of oxygen to the temperature and oxygen partial pressure using the following equation^36^,

2 $${\mu }_{O}=\frac{1}{2}({E}_{{O}_{2}}^{DFT}+({G}_{{O}_{2}}^{0}(T)-{G}_{{O}_{2}}^{0}(0K))+{k}_{B}T\,\mathrm{ln}(\frac{{P}_{{O}_{2}}}{{p}^{0}}))$$

where ${E}_{{O}_{2}}^{DFT}$ is the energy of one O_2_ molecule obtained by DFT calculations. ${G}_{{O}_{2}}^{0}$ is the Gibbs free energy of the gaseous O_2_ phase under the standard pressure ${p}^{0}$ as a function of temperature, which is estimated by assuming an ideal gas on the basis of experimental results^37^.

### Molecular dynamics simulation to evaluate the tilted grain boundary of Σ3 (2–1–1) = (1–21)

Molecular dynamics (MD) simulations were performed using a Born-like description of the ionic crystal lattice^38^. The long-range Coulombic interactions were summed via the Ewald method^39^, whereas the short-range interactions were described using the parameterized Buckingham pair potentials^40^. The latter were summed to the cut-off value of 10.5 Å, beyond which the influence of the potential was considered negligible. The lattice energy is defined as

3 $${E}_{L}=\sum _{i}\sum _{j > i}[\frac{{q}_{i}{q}_{j}}{4\pi {\varepsilon }_{0}{r}_{ij}}+{A}_{ij}\exp (\frac{-{r}_{ij}}{{\rho }_{ij}})-(\frac{{C}_{ij}}{{r}_{ij}^{6}})]$$

where ${r}_{ij}$ is the separation between ions *i* and *j*, *q*_*i*_ and *q*_*j*_ are the ion charges, and ${\varepsilon }_{0}$ is the permittivity of the free space. The Buckingham potential parameters, ${A}_{ij}$, ${\rho }_{ij}$, and ${C}_{ij}$, were specific to the pairs of interacting species. The simulation parameters are listed in Table S1 ^41^.

The DL POLY simulation package^42^ was used for all MD calculations, and the corresponding time step was equal to 1 fs. First, the initial models were equilibrated for 20 000 time steps (20 ps) in the isothermal isobaric (NPT) ensemble at a temperature of 300 K. During this initial period, the volume of the cell was allowed to relax with time. The Nosé-Hoover thermostat and barostats^43,44^ were used to control the temperature and pressure, respectively. Afterwards, the temperature was elevated from 700 to 1700 K at a rate of 10 K per 5 ps; and the NPT dynamic simulations were conducted for 100 ps, during which the cell angles were allowed to relax. After equilibration at 1700 K, the obtained structure was cooled to a desired temperature, which allowed the lattice to fully equilibrate in a shorter simulation time compared to that required for directly heating the system to the target temperature. Cooling was performed via an iterative procedure, which involved decreasing the temperature in steps of 10 K accompanied by dynamic simulations with durations of 5 ps. For each studied temperature, MD simulations containing 500 000 time steps (500 ps) were performed using the constant volume and temperature (NVT) ensemble, in order to obtain statistical information about diffusion rates. The bulk Li_5_La_3_Nb_2_O_12_ calculations were performed using the 3 × 3 × 3 unit cell superstructure with cubic symmetry and containing 4752 atoms.

The GB energy, ${\gamma }_{GB}$, is defined as

4 $${\gamma }_{GB}=\frac{1}{2A}({E}_{GB}-N{E}_{bulk})$$

where *A* is the GB area, *E*_GB_ is the lattice energy of the GB model, *E*_bulk_ is the lattice energy per atom, and *N* is the number of atoms in the particular GB model. To investigate the ionic transport properties of a particular structure, the mean square displacement (MSD) of the ions was monitored as a function of time at different temperatures. For a system with *N* ions, the MSD of ion *i* at position *r*_*i*_(*t* + *t*_0_) and time *t* with respect to its initial position *r*_*i*_(*t*_0_) is defined as

5 $$\langle {r}^{2}(t)\rangle =\langle \frac{1}{N}\sum _{i=0}^{N}{({r}_{i}(t+{t}_{0})-{r}_{i}({t}_{0}))}^{2}\rangle .$$

The Li diffusion coefficient, *D*, can be calculated from the MSD slopes plotted against time^45^:

6 $$\langle {|{r}_{i}(t+{t}_{0})-{r}_{i}({t}_{0})|}^{2}\rangle =6Dt+B$$

where B is the atomic displacement parameter related to thermal vibrations. The Li ionic conductivity, ${\sigma }_{Li}$, can be calculated by using the Nernst-Einstein equation^46^:

7 $${\sigma }_{Li}={c}_{Li}{({z}_{Li}F)}^{2}\frac{{D}_{Li}}{RT}$$

where ${c}_{Li}$ is the Li carrier density, ${z}_{Li}$ is the Li charge, *F* is Faraday’s constant, *R* is the gas constant, and *T* is the temperature. The Li ionic conductivity values were obtained in the temperature range of 700–1700 K.

### Growth of Li_5_La_3_Nb_2_O_12_ crystal layer on LiCoO_2_ ceramics

A thin Nb film was deposited on the LiCoO_2_ ceramics by RF-driven Ar sputtering under 0.67 Pa. The back pressure, input power, and flow rate of Ar gas were controlled to be 2.0 × 10^−3^ Pa, 300 W, and 3.0 sccm, respectively. The 1 mm thick LiCoO_2_ ceramics with sintering density of >98% was gently placed on the mixture of La_2_O_3_ (0.305 g) LiOH·H_2_O (0.295 g) and then heated to 500 °C at a rate of 500 °C·h^−1^ in an electric furnace. After maintaining this temperature for a designated period, the crucible was cooled to 300 °C at the rate of 100 °C·h^−1^, and then naturally cooled to room temperature in the furnace. The substrate was separated from the mixture by washing with warm water for three times to remove the remaining flux. Finally, the substrate was dried at 60 °C under air for 10 h.

## Electronic supplementary material

supplementary information

## References

[1] Robinson AL, Janek J (2014). Solid-state batteries enter EV fray. MRS Bull..

[2] Kato Y (2016). High-power all-solid-state batteries using sulfide superionic conductors. Nature Energy.

[3] Bates JB (2000). Preferred orientation of polycrystalline LiCoO_2_ film. J. Electrochem. Soc..

[4] Iriyama Y, Kako T, Yada C, Abe T, Ogumi Z (2005). Charge transfer reaction at the lithium phosphorus oxynitride glass electrolytelithium cobalt oxide thin film interface. Solid State Ionics.

[5] Kato T (2016). Preparation of thick-film electrode-solid electrolyte composites on Li_7_La_3_Zr_2_O_12_ and their electrochemical properties. J. Power Sources.

[6] Kobayashi E, Plashnitsa LS, Doi T, Okada S, Yamaki J (2010). Electrochemical properties of Li symmetric solid-state cell with NASICON-type solid electrolyte and electrodes. Electrochem. Comm..

[7] Aboulaich A (2011). A new approach to develop safe all-inorganic monolithic Li-ion battery. Adv. Energy Mater..

[8] Ohta S (2013). All-solid-state lithium ion battery using garnet-type oxide and Li_3_BO_3_ solid electrolytes fabricated by screen-printing. J. Power Sources.

[9] Huang M, Dumon A, Nan C-W (2012). Effect of Si, In and Ge doping on high ionic conductivity of Li_7_La_3_Zr_2_O_12_. Electrochem. Comm..

[10] Narayanan S, Epp V, Wilkening M, Thangadurai V (2012). Macroscopic and microscopic Li^+^ transport parameters in cubic garnet-type “Li_6.5_La_2.5_Ba_0.5_ZrTaO_12_” as probed by impedance spectroscopy and NMR. RSC Advances.

[11] Murugan R, Ramakumar S, Janani N (2011). High conductive yttrium doped Li_7_La_3_Zr_2_O_12_ cubic lithium garnet. Electrochem. Comm..

[12] Ohta S, Kobayashi T, Asaoka T (2011). High lithium ionic conductivity in the garnet-type oxide L_i7_ − XL_a_3(Z_r2_ − X, N_*b*_X)_O1_2 (X = 0–2). J. Power Sources.

[13] Li Y (2012). Ionic distribution and conductivity in lithium garnet Li_7_La_3_Zr_2_O_12_. J. Power Sources.

[14] Narayanan S, Ramezanipour F, Thangadurai V (2012). Enhancing Li ion conductivity of garnet-type Li_5_La_3_Nb_2_O_12_ by Y- and Li-codoping: synthesis, structure, chemical stability, and transport properties. J. Phys Chem. C..

[15] Kotobuki M, Munakata H, Kanamura K, Sato Y, Yoshida T (2010). Compatibility of Li_7_La_3_Zr_2_O_12_ Solid Electrolyte to All-Solid-State Battery Using Li Metal Anode. J. Electrochem. Soc..

[16] Kotobukia M, Kanamura K, Sato Y, Yamamoto K, Yoshida T (2012). Electrochemical properties of Li_7_La_3_Zr_2_O_12_ solid electrolyte prepared in argon atmosphere. J. Power Sources.

[17] Tan J, Tiwari A (2012). Fabrication and Characterization of Li_7_La_3_Zr_2_O_12_ Thin Films for Lithium Ion Battery. ECS Solid State Lett..

[18] Kim S, Hirayama M, Taminato S, Kanno R (2013). Dalton Trans..

[19] Kalita DJ, Lee KSH, Lee S, Ko DH, Yoon YS (2012). Ionic conductivity properties of amorphous Li–La–Zr–O solid electrolyte for thin film batteries. Solid State Ionics.

[20] Tadanaga K (2015). Preparation of lithium ion conductive Al-doped Li_7_La_3_Zr_2_O_12_ thin films by a sol-gel process. J. Power Sources.

[21] Inada, R., Okada, T., Bando, A., Tojo, T. & Sakurai, Y. Properties of garnet-type Li_6_La_3_ZrTaO_12_ solid electrolyte films fabricated by aerosol deposition method. *Progress in Natural Science: Materials International*, in press.

[22] Hayashi K, Noguchi H, Sato I (1986). New Phse in the La_2_O_3_-Li_2_O-Nb_2_O_5_ system. Mater. Res. Bull..

[23] Yoda T (2015). Flux growth of patterned LiCoO_2_ crystal arrays directly on Pt substrate in molten LiNO_3_. RSC Adv..

[24] Zettsu N (2016). Growth of hollow-structured LiMn_2_O_4_ crystals starting from Mn metal in molten KCl through the microscale Kirkendall effect. CrystEngComm.

[25] Mizuno Y (2013). Environmentary Friendly Flux Growth of High-Quality, Idiomorphic Li_5_La_3_Nb_2_O_12_ crystals. Cryst. Growth. Des..

[26] Zettsu N (2015). Flux growth of hexagonal cylindrical LiCoO2 crystals surrounded by Li-ion conducting preferential facets and their electrochemical properties studied by single particle measurements. J. Mater. Chem. A.

[27] Shiiba, H. *et al*. Insight for lithium ion conduction behavior of grain boundaries of cubic Li_7_La_3_Zr_2_O_12_ with a garnet framework, submitted.

[28] Thangadurai V, Kaack H, Weppner WJF (2003). Novel fast lithium ion conduction in garnet-type Li_5_La_3_M_2_O_12_ (M = Nb, Ta). J. Am. Ceram. Soc..

[29] Bitzer M, Gestel TV, Uhlenbruck S, Buchkremer H-P (2016). Thin Solid Films.

[30] Kresse G, Furthmuller J (1996). Efficient iterative schemes for *ab initio* total-energy calculations using a plane-wave basis set. Phys. Rev. B.

[31] Kresse G, Furthmuller J (1996). Efficiency of *ab-initio* total energy calculations for metals and semiconductors using a plane-wave basis set. Comput. Mater. Sci..

[32] Perdew JP (2008). Restoring the Density-Gradient Expansion for Exchange in Solids and Surfaces. Phys. Rev. Lett..

[33] Blochl PE (1994). Projector augmented-wave method. Phys. Rev. B.

[34] Hautier G, Ong SP, Jain A, Moore CJ, Ceder G (2012). Accuracy of density functional theory in predicting formation energies of ternary oxides from binary oxides and its implication on phase stability. Phys. Rev. B.

[35] Wang L, Maxisch T, Ceder G (2006). Oxidation Energies of Transition Metal Oxides within the GGA + U Framework. Phys. Rev. B.

[36] Koyama Y, Arai H, Tanaka I, Uchimoto Y, Ogumi Z (2012). Defect Chemistry in Layered Li*M*O_2_ (*M* = Co, Ni, Mn, and Li_1/3_Mn_2/3_) by First-Principles Calculations. Chem. Mater..

[37] Stewart RB, Jacobsen RT, Wagner W (1991). Thermodynamic Properties of Oxygen from the Triple Point to 300 K with Pressures to 80 MPa. J. Phys. Chem. Ref. Data..

[38] Born M, Mayer JE (1932). Zur Gittertheorie der lonenkristalle. Z. Phys..

[39] Ewald PP (1921). Die Berechnung optischer und elektrostatischer Gitterpotentiale. Ann. Phys..

[40] Buckingham RA (1938). The Classical Equation of State of Gaseous Helium, Neon and Argon. Proc. R. Soc. London, Ser. A.

[41] Jalem R (2015). Insights into the Lithium-Ion Conduction Mechanism of Garnet-Type Cubic Li_5_La_3_Ta_2_O_12_ by *ab-Initio* Calculations. Chem. Mater..

[42] Todorov, I. T. & Smith, W. The DLPOLY 4.01.1 User Manual, Daresbury Laboratory, UK.

[43] Nose S (1984). A unified formulation of the constant temperature molecular dynamics methods. J. Chem. Phys..

[44] Hoover WG (1985). Canonical dynamics: Equilibrium phase-space distributions. Phys. Rev. A: At., Mol., Opt. Phys..

[45] Gillan MJ (1985). The simulation of superionic materials. Physica B (Amsterdam).

[46] Zahn D (2011). Molecular dynamics simulation of ionic conductors: perspectives and limitations. J. Mol. Model..

