# Supplementary material for: Thin and Dense Solid-solid Heterojunction Formation Promoted by Crystal Growth in Flux on a Substrate
Source: Sci Rep. 2018 Jan 8;8:96. doi: 10.1038/s41598-017-18250-9 (PMC5758689; doi:10.1038/s41598-017-18250-9)
Supplement: Supplementary file 1 — supplementary information [file 41598_2017_18250_MOESM1_ESM.pdf]

Supplementary Materials

Thin and Dense Solid-solid Heterojunction Formation Promoted by Crystal Growth in Flux on a Substrate

Nobuyuki Zettsu[1,2,§], Hiromasa Shiiba[2, §], Hitoshi Onodera[2], Kazune Nemoto[2], Takeshi Kimijima[2], Kunio Yubuta[3], Masanobu Nakayama[4,5], and Katsuya Teshima[1,2,*]

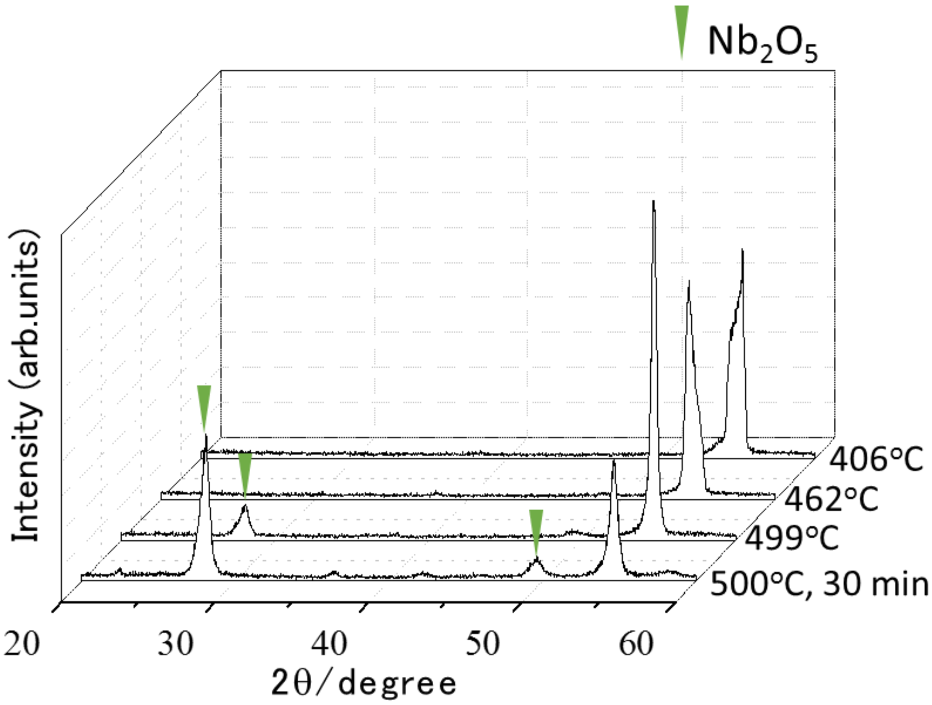

Fig. S1. *In-situ* XRD patterns of Nb substrate under heating in air.

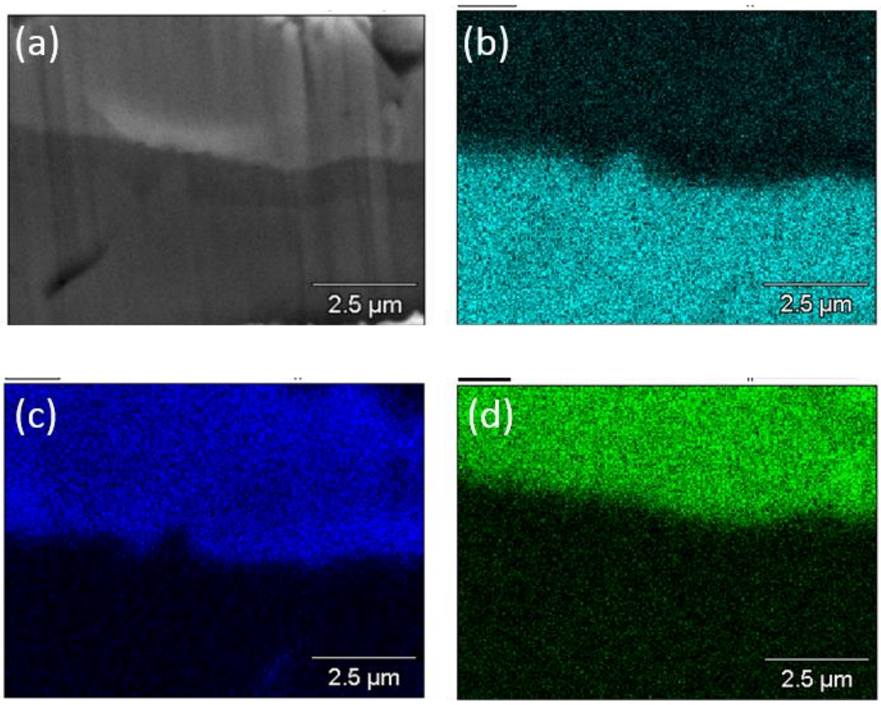

Fig. S2. Cross-sectional SEM-EDS elemental mappings of $Li_5La_3Nb_2O_{12}$ crystal layer on $LiCoO_2$ ceramic after heating at 500 °C for 10 h: (a) SEM image, (b) Co, (c) Nb, and (d) La.

Table S1. Parameters of the Buckingham inter-ionic potentials.

Interaction	A_{ij} [eV]	P_{ij} [Å]	C_{ij} [eV Å^6]
$Li^{0.7+}$ - $O^{1.4-}$	876.86	0.2433	0
$La^{2.1+}$ - $O^{1.4-}$	14509.63	0.2438	30.83
$Nb^{3.5+}$ - $O^{1.4-}$	2325.372	0.2913	0
$O^{1.4-}$ - $O^{1.4-}$	4869.99	0.2402	27.22
